# Restoration of Functional Oral Intake After Proximal Gastrectomy Using an Individualized Risk‐Adapted Multidisciplinary Approach: A Case Report

**DOI:** 10.1002/ccr3.73459

**Published:** 2026-09-09

**Authors:** Shogo Ito, Hirokazu Takada, Kazuhiro Shibata, Hidekazu Yamamoto

**Affiliations:** ^1^ Private Practice Kamishihoro Dental Clinic Kamishihoro Hokkaido Japan; ^2^ Department of Oral Biochemistry and Molecular Biology, Graduate School of Dental Medicine Hokkaido University Sapporo Japan

**Keywords:** chronic malnutrition, dental implant rehabilitation, functional oral intake, gastrectomy, iron deficiency anemia, multidisciplinary management

## Abstract

Dental rehabilitation restored functional oral intake after gastrectomy, followed by long‐term improvement in BMI and stabilization of hematologic, iron‐status, and metabolic parameters. A risk‐adapted multidisciplinary approach combining medical optimization and oral rehabilitation may benefit selected patients with post‐gastrectomy malnutrition.

## Key Clinical Message

Restoration of functional oral intake after dental rehabilitation may contribute to improvement in BMI and long‐term stabilization of hematologic and metabolic parameters following gastrectomy. Evaluation of oral function should be incorporated into multidisciplinary management together with anthropometric, hematologic, inflammatory, and metabolic assessments.

## 1 Introduction

Gastric cancer remains a major global health burden, ranking as the fifth most commonly diagnosed cancer worldwide. In 2020, more than 1.1 million new cases and approximately 770,000 deaths were reported globally [1].

Although advances in surgical treatment have improved survival outcomes, long‐term complications after gastrectomy remain clinically significant. Postgastrectomy syndrome includes dumping syndrome, nutritional impairment, iron deficiency anemia, bone metabolism disorders, and Roux‐en‐Y–related symptoms, all of which may substantially reduce patients' quality of life [2].

In particular, proximal gastrectomy may be associated with impaired absorption of iron and vitamin B12 [3].

Furthermore, impaired oral function, including tooth loss and occlusal insufficiency, has been associated with poor nutritional status in older adults [4, 5]. When masticatory dysfunction coexists with malabsorption, chronic malnutrition may become refractory to conventional supplementation alone.

Herein, we report the long‐term clinical course of a patient with both post‐gastrectomy malabsorption and impaired mastication who was managed using an individualized, staged multidisciplinary approach combining medical correction of iron deficiency with restoration of functional oral intake.

## 2 Case Report

A 75‐year‐old woman was referred for evaluation of masticatory difficulty in the left mandibular molar region. At 59 years of age, she had undergone proximal gastrectomy (approximately three‐quarters resection) for Stage III gastric cancer, followed by 2 years of adjuvant chemotherapy. No recurrence had been observed.

### 2.1 Medical Diagnoses

Post‐gastrectomy status (16 years after surgery for Stage III gastric cancer).

Iron deficiency anemia secondary to gastrectomy‐induced malabsorption.

Hypoalbuminemia.

Chronic malnutrition.

At presentation, hemoglobin (Hb) was 8.5 g/dL, serum albumin (ALB) was 3.5 g/dL, ferritin was 5.2 ng/mL and mean corpuscular volume (MCV) was 79 fL. Following dietary intervention by a registered dietitian, Hb increased to 9.7 g/dL and ALB to 3.7 g/dL; however, ferritin showed only a minimal increase to 5.3 ng/mL, while MCV increased to 81 fL. Hb, ALB, and ferritin remained below the respective laboratory reference ranges. The most notable laboratory finding was the persistent iron‐deficiency pattern despite dietary intervention. Ferritin remained almost unchanged at 5.3 ng/mL, suggesting that dietary management alone was insufficient to replenish iron stores in the setting of post‐gastrectomy malabsorption.

### 2.2 Dental Findings and Diagnoses

Oral examination revealed reduced occlusal support in the left posterior region and difficulty chewing solid foods. Panoramic radiography demonstrated furcation involvement of tooth #37 with mild mobility.

The dental diagnoses were:

Stage II, Grade B periodontitis.

Furcation involvement of tooth #37 (Hamp classification Grade I).

Reduced posterior occlusal support with impaired masticatory function.

These findings were considered to contribute to impaired functional oral intake.

### 2.3 Treatment Strategy

The treatment strategy consisted of a staged multidisciplinary approach. First, patient‐specific clinical targets were established prior to invasive intervention. Under physician supervision, intravenous iron (40 mg ferric preparation) and vitamin B12 (500 μg) were administered six times over 2 months. After intravenous iron and vitamin B12 administration, Hb increased to 11.3 g/dL, ferritin to 40.0 ng/mL, and MCV to 90 fL, indicating correction of the iron‐deficiency pattern. ALB increased to 3.9 g/dL. Based on the overall clinical and laboratory assessment, invasive dental treatment was considered appropriate for this individual patient.

Second, oral surgical intervention and implant placement were performed to restore functional oral intake. Teeth #34, #35, and #37 were extracted, followed by implant placement. No postoperative complications were observed.

From the third year onward, implant surgical procedures were performed in a staged manner in the left mandibular molar region, right maxillary molar region, and right mandibular molar region, while carefully monitoring changes in hematologic parameters. A provisional prosthesis was placed in the fourth year, and treatment was completed with placement of the final prosthesis at 4 years and 3 months.

**FIGURE 1:**
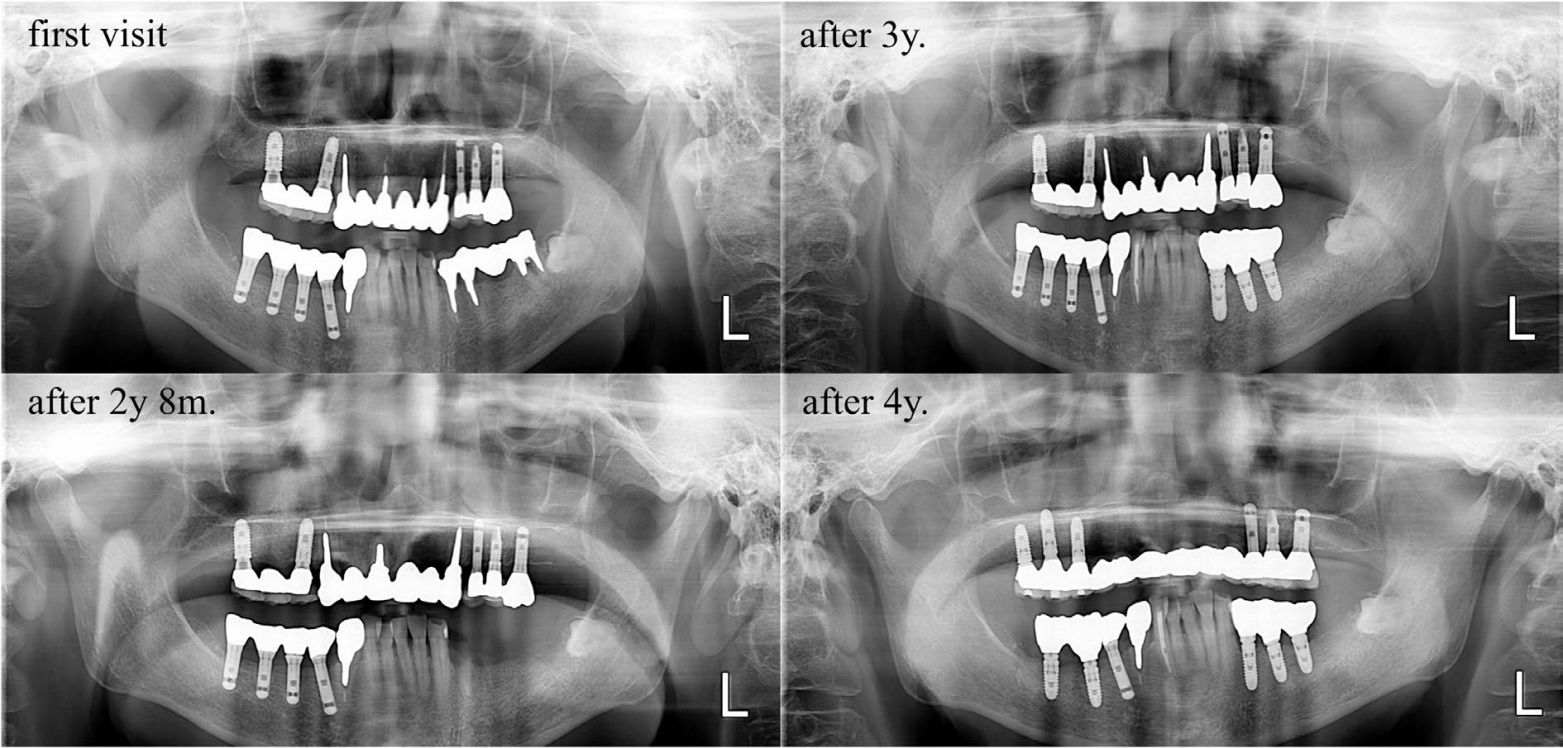
Serial panoramic radiographs from baseline, surgical intervention, and prosthetic completion.

**FIGURE 2:**
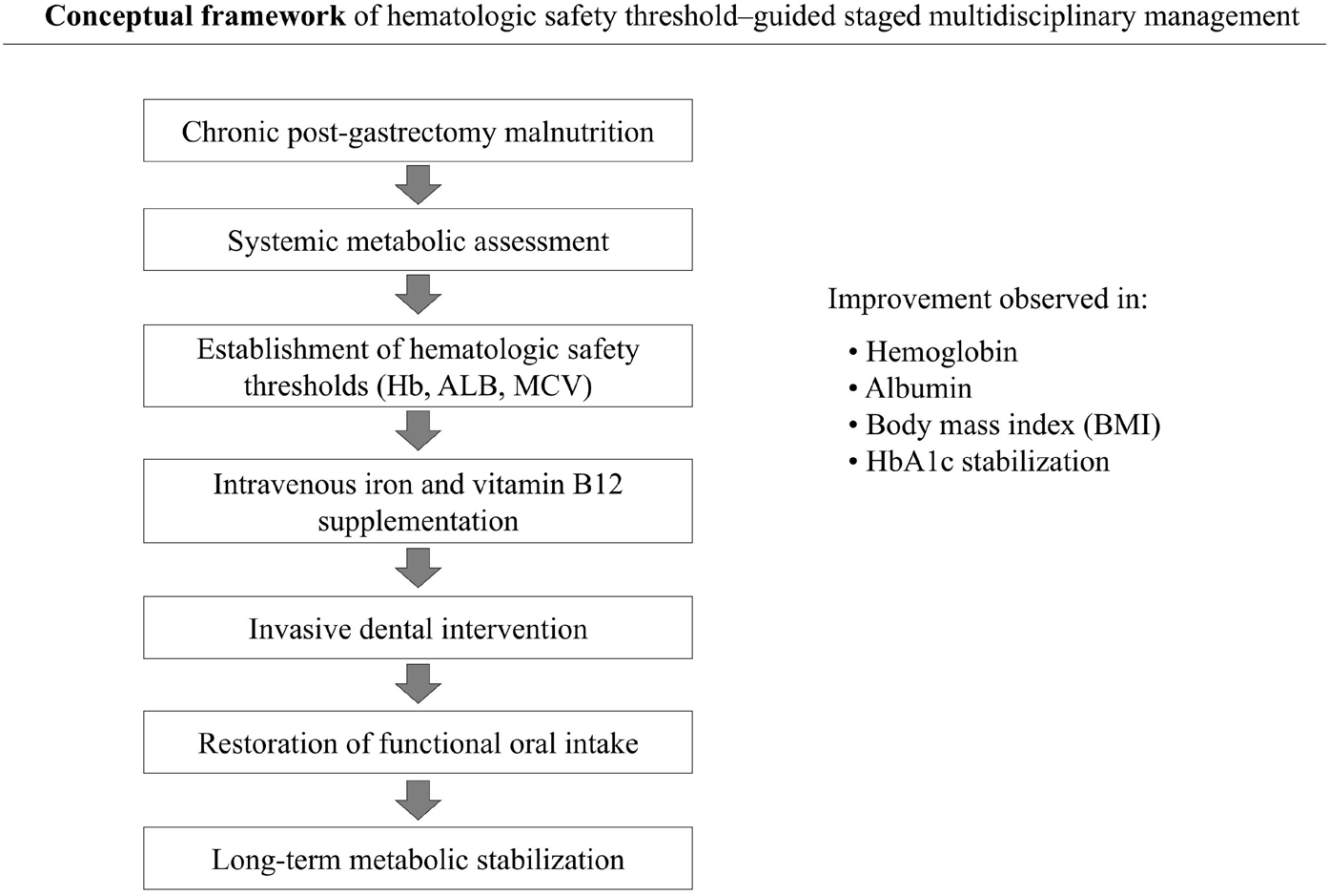
Conceptual framework of an individualized, risk‐adapted staged multidisciplinary approach. A staged strategy beginning with individualized systemic and laboratory assessment prior to invasive intervention, followed by dental rehabilitation and restoration of functional oral intake. Improvement in BMI and long‐term stabilization of hematologic, iron‐status, and metabolic parameters were observed during follow‐up after functional rehabilitation.

**FIGURE 3:**
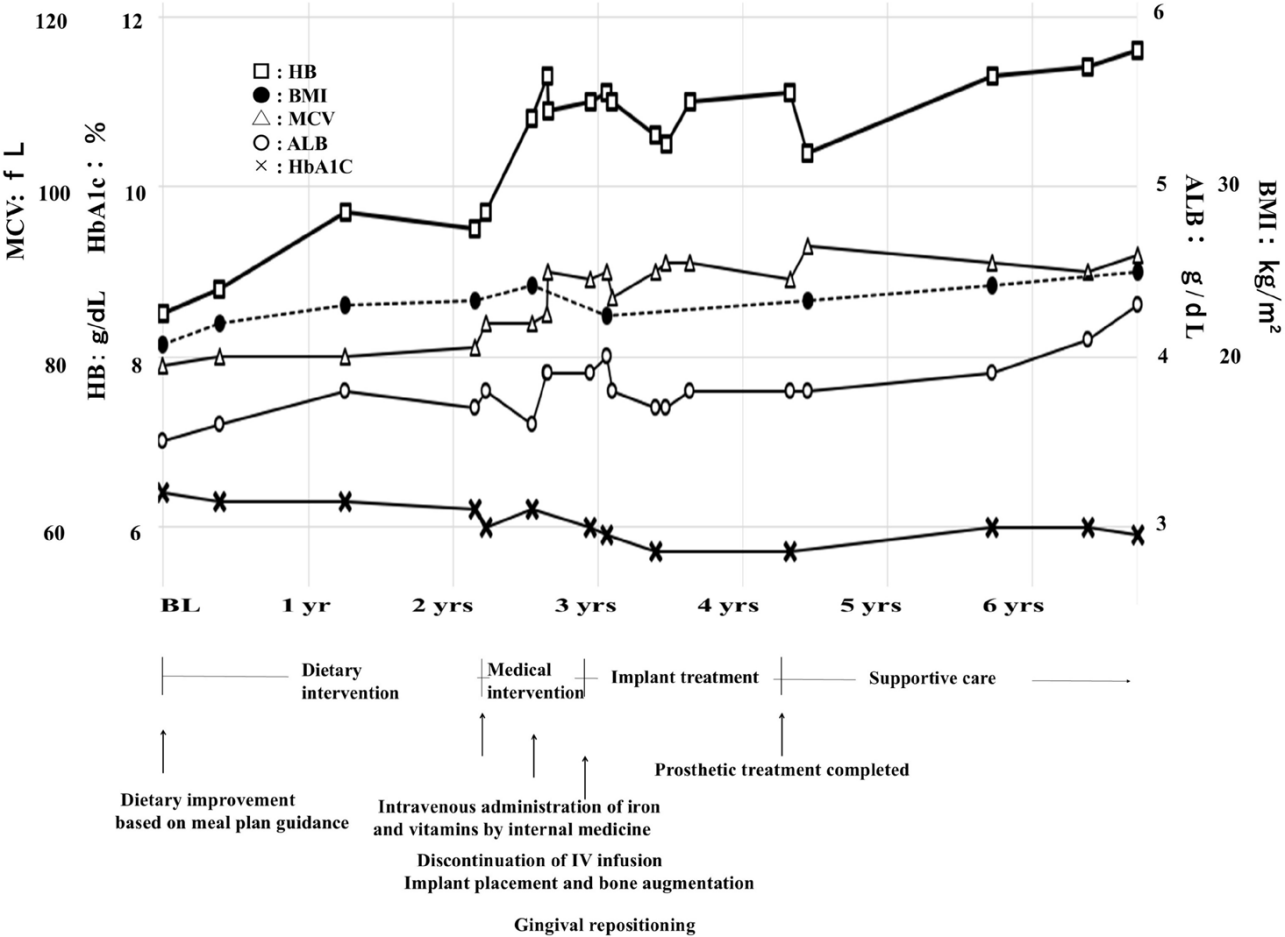
Longitudinal changes in hematologic and metabolic parameters. Temporal changes in Hb, ALB, BMI, MCV, and HbA1c during staged intervention and follow‐up.

**FIGURE 4:**
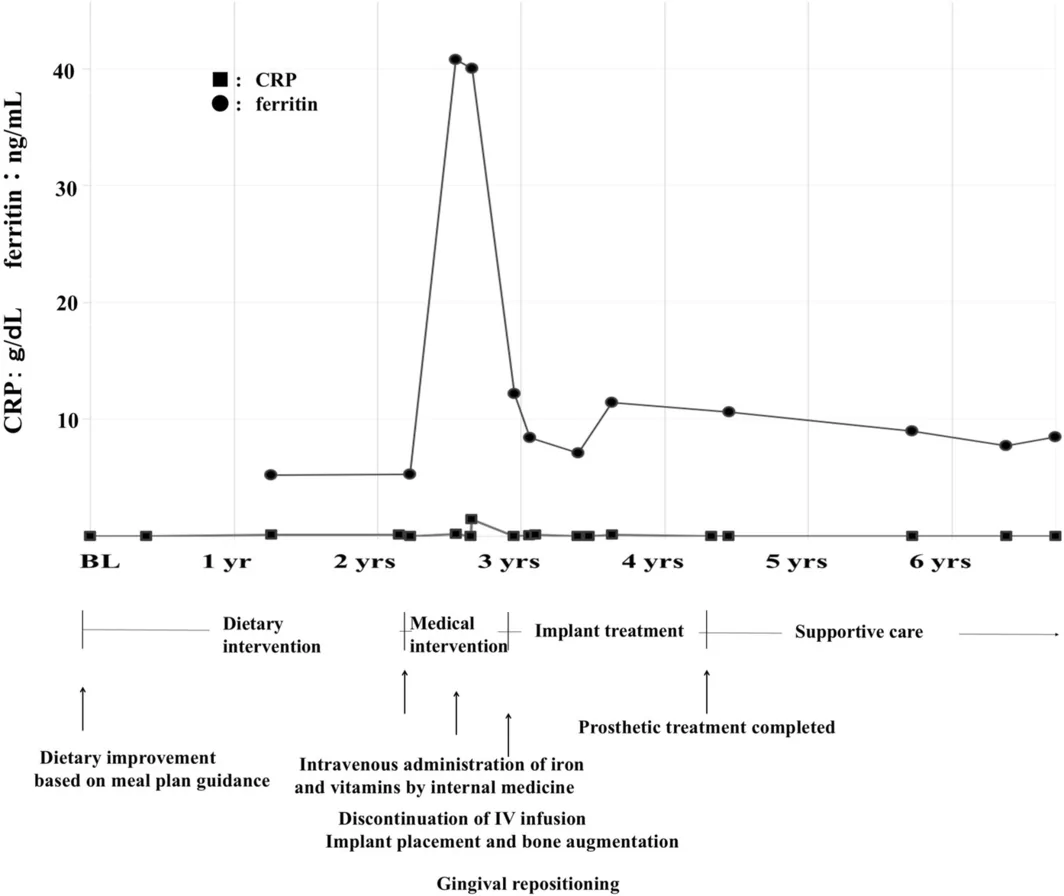
Changes in inflammatory and iron‐status markers. CRP levels remained within or near the normal range during follow‐up. Ferritin remained relatively stable at approximately 10 ng/mL, slightly below the diagnostic cutoff of 12 ng/mL, without evidence of a marked further decline in iron stores.

During follow‐up after restoration of functional oral intake, Hb increased from a nadir of 8.5 g/dL to 11.6 g/dL, ALB from 3.5 to 4.3 g/dL, and BMI from 20.3 to 22.6 kg/m^2^, while HbA1c decreased from 6.5% to 5.8%. Ferritin showed little change after dietary intervention (5.2 → 5.3 ng/mL). After completion of iron supplementation, it temporarily decreased but subsequently stabilized at approximately 10 ng/mL. In the absence of elevated CRP levels, ferritin was unlikely to have been substantially influenced by persistent systemic inflammation. Although ferritin remained slightly below the diagnostic cutoff of 12 ng/mL, no marked further decline in iron stores was observed during follow‐up. The increase in BMI and the stabilization of hematologic, iron‐status, and metabolic parameters were maintained for more than 3 years without additional pharmacologic therapy (Figures 1, 2, 3, 4).

The reference values and clinical criteria used for interpretation were as follows: hemoglobin, 12.1–14.5 g/dL for adult women; serum albumin, ≥ 3.9 g/dL; and C‐reactive protein, ≤ 0.30 mg/dL, according to the 2026 criteria of the Japan Society of Ningen Dock and Preventive Medical Care [6]. A ferritin level < 12 ng/mL was interpreted as indicating iron deficiency in accordance with the criteria of the Japanese BioIron Society [7]. The reference ranges provided by our clinical laboratory were 80–101 fL for mean corpuscular volume and 4.6%–6.2% for HbA1c.

## 3 Discussion

The clinical significance of this case lies in the long‐term observation of an individualized multidisciplinary approach in a patient with both post‐gastrectomy malabsorption and impaired mastication. Iron deficiency was first corrected medically, followed by staged dental rehabilitation. After restoration of oral function, improvement in BMI and stabilization of hematologic, iron‐status, and metabolic parameters were observed for more than 3 years.

The decision to proceed with invasive dental treatment was based on an individualized assessment conducted in collaboration with the treating physician. This assessment included improvement in iron‐deficiency anemia, absence of active inflammation, overall clinical stability, the elective and invasive nature of the planned treatment, and the availability of less invasive alternatives such as removable prostheses. Hb ≥ 10 g/dL, ALB ≥ 3.9 g/dL, and CRP ≤ 0.3 mg/dL were used as patient‐specific clinical targets and were not regarded as validated or universally applicable thresholds for implant surgery.

Chronic malnutrition after gastrectomy remains a persistent clinical challenge, particularly in older adults. Altered gastrointestinal anatomy may impair iron absorption and contribute to persistent iron‐deficiency anemia. Although hypoalbuminemia may also be observed, serum albumin is influenced by inflammation, disease severity, hepatic function, and fluid status and should not be interpreted as a direct marker of nutritional intake or protein‐energy malnutrition [8]. Nevertheless, lower preoperative albumin levels have been associated with an increased risk of postoperative complications, including when albumin is analyzed as a continuous variable [9]. Therefore, in this case, ALB was used as one component of individualized perioperative risk assessment rather than as a nutritional marker or an absolute criterion for proceeding with surgery.

Ferritin was also not used as a strict treatment threshold but was interpreted together with improvement in iron‐deficiency anemia, inflammatory status, and the overall clinical course. Standardized laboratory criteria for implant surgery in patients with chronic malnutrition have not been established; therefore, the laboratory values used in this case should not be generalized to other patients. Rather, this case illustrates the importance of individualized systemic assessment and multidisciplinary consultation when invasive dental treatment is considered in medically compromised patients.

At presentation, the patient's BMI was 20.3 kg/m^2^, which was below the GLIM low‐BMI criterion of 22 kg/m^2^ for individuals aged 70 years or older [10]. Reduced intake related to masticatory dysfunction and impaired nutrient absorption after gastrectomy were also present as etiologic factors. However, detailed longitudinal data on unintentional weight loss, actual energy intake, and muscle mass were unavailable. Therefore, a definitive retrospective diagnosis and severity classification of malnutrition using the full GLIM criteria could not be performed.

Although causality cannot be established from a single case, restoration of oral function may have been one of several factors associated with the subsequent clinical course. Other potential contributors included dietary counseling, intravenous iron and vitamin B12 supplementation, natural physiological variation, changes in appetite or food selection, and unrecorded lifestyle or medical factors. Therefore, the association between restoration of oral function and subsequent changes in laboratory parameters should be interpreted cautiously.

Mastication influences digestive physiology, including insulin and incretin responses. Increased chewing has been reported to improve postprandial glucose metabolism [11]. In addition, masticatory inefficiency caused by diminished occlusal support has been associated with poorer glycemic control in patients with Type 2 diabetes [12]. In this patient, restoration of posterior occlusal support and functional mastication was followed by improvement in BMI and stabilization of hematologic and metabolic parameters. These findings are compatible with, but do not establish, an association between oral functional capacity and systemic metabolic status.

Dental rehabilitation improved masticatory function and enabled the patient to consume regular meals, including solid foods that had previously been difficult to chew. This suggests that impaired mastication may contribute to reduced oral intake in some patients after gastrectomy and that restoration of oral function may support improved intake capacity.

Prosthetic treatment options for tooth loss include removable dentures and fixed bridges. These approaches have the advantage of avoiding surgical intervention, but they may not restore masticatory function to the same extent as implant‐supported rehabilitation. Implant treatment may provide greater functional recovery; however, it requires surgery and a relatively prolonged treatment period. Therefore, in medically compromised patients, treatment invasiveness, systemic condition, expected functional benefit, and less invasive alternatives should be carefully weighed.

In this case, CRP remained within or near the normal range during follow‐up. Although ALB increased without a concurrent elevation in CRP, this change was not interpreted as direct evidence of improved nutritional status because serum albumin is influenced by systemic inflammation, disease severity, and fluid balance [8]. Ferritin remained relatively stable at approximately 10 ng/mL, slightly below the diagnostic cutoff of 12 ng/mL, without evidence of a marked further decline in iron stores.

Epidemiological studies have shown that the number of functional teeth predicts all‐cause mortality more strongly than the number of present teeth, suggesting that oral functional capacity is closely associated with systemic health [13]. In addition, a previous case report described improvement in iron‐deficiency anemia following implant therapy [14]. Together, these findings support the clinical relevance of evaluating oral function as one component of multidisciplinary management in selected patients with chronic nutritional impairment after gastrectomy.

## 4 Conclusions

Restoration of functional oral intake following individualized systemic assessment and medical optimization may represent a clinically relevant component of multidisciplinary management in selected patients with chronic malnutrition after gastrectomy. Although further studies involving larger cohorts are required, integration of systemic evaluation and functional rehabilitation may represent a rational strategy in similar clinical settings.

These findings suggest that assessment of oral function may play an important role in the multidisciplinary management of chronic malnutrition following gastrectomy.

## Author Contributions

**Shogo Ito:** writing – original draft, conceptualization. **Hirokazu Takada:** validation, formal analysis, writing – review and editing. **Hidekazu Yamamoto:** investigation, data curation, writing – review and editing, conceptualization. **Kazuhiro Shibata:** writing – review and editing, supervision.

## Funding

The authors have nothing to report.

## Ethics Statement

This case report was conducted in accordance with the Declaration of Helsinki.

## Consent

Written informed consent was obtained from the patient for publication of this report and accompanying images.

## Conflicts of Interest

The authors declare no conflicts of interest.

## Data Availability

Data sharing not applicable to this article as no datasets were generated or analysed during the current study.
